# An investigation of heavy metals in edible bird’s nest from Indonesia using inductively coupled plasma mass spectrometry

**DOI:** 10.14202/vetworld.2022.509-516

**Published:** 2022-02-27

**Authors:** Dede Sri Wahyuni, Hadri Latif, Mirnawati B. Sudarwanto, Chaerul Basri, Daniel Thong

**Affiliations:** 1 Veterinary Public Health Study Program, Faculty of Veterinary Medicine, IPB University, Bogor, Indonesia; 2 Soekarno Hatta Agricultural Quarantine Center, Agricultural Quarantine Agency, Agricultural Quarantine Building Soekarno Hatta International Airport, Pajang, Benda, Tangerang City, Banten 15126, Indonesia; 3 Department of Animal Infectious Diseases and Veterinary Public Health, Faculty of Veterinary Medicine, IPB University, Bogor, Indonesia; 4 Chairperson of the Farmers Association Edible Bird Nest Nusantara, Tangerang, Banten, Indonesia

**Keywords:** edible bird nests, heavy metal, swiftlet farmhouse, washing

## Abstract

**Background and Aim:**

In 2020, Indonesia, which has the highest global production of edible bird’s nest (EBNs), exported up to 1312.5 tons of this product at a value of USD 540.4 million. Recently, food safety aspects related to EBNs, including contamination with heavy metals, have become a serious concern. However, data on the presence and concentration of heavy metals in EBNs in Indonesia are not yet available. This study aimed to determine and compare the presence and concentrations of arsenic (As), mercury (Hg), lead (Pb), cadmium (Cd), and tin (Sn) in EBNs originating from several primary Indonesian islands. The study also analyzed the effect of washing on the heavy metal content in EBNs.

**Materials and Methods:**

A study on 44 swiftlet farmhouses (SFHs) was conducted to determine the concentrations of heavy metals in EBNs. The number of samples from the SFHs was allocated proportionally to the main EBN-producing islands in Indonesia, that is, Kalimantan, Sumatera, Sulawesi, and Java (22, 13, 7, and 2, respectively). The concentrations of the above five elements in the samples before washing (raw–unclean EBNs) and after washing (raw–clean EBNs) were determined by inductively coupled plasma mass spectrometry. Washing was conducted according to the general procedures at an EBN processing plant.

**Results:**

The raw–unclean EBNs from the four islands contained As, Pb, Cd, and Sn at varying concentrations. However, Hg was not detected in the raw–unclean EBN samples from Sulawesi. The raw–unclean EBNs from Kalimantan had lower concentrations of Pb and Cd compared with the other islands. The concentrations of As, Pb, Cd, and Sn in the EBNs decreased significantly after washing with clean water.

**Conclusion:**

Heavy metals (As, Hg, Pb, Cd, and Sn) were detected at a low level in most of the raw–unclean EBNs originating from the main Indonesian island where they were produced. The concentrations of all the heavy metals reviewed in the raw–unclean EBNs samples decreased significantly after washing.

## Introduction

Indonesia has the highest production of edible bird nests (EBNs) worldwide (85%), followed by Malaysia and Thailand [1]. In 2020, Indonesia exported up to 1,312.5 tons of EBNs at a value of USD540.4 million. The demand for EBN exports in Indonesia increased by 4.27% in 2020 compared with 2019 [2]. In Indonesia, EBNs are produced mainly by two swiftlet species, that is, the white-nest swiftlet (*Aerodramus fuciphagus*) and the black-nest swiftlet (*Aerodramus maximus*) [3]; however, only EBNs from *A. fuciphagus* are harvested commercially [4]. EBNs are created from the secretion of saliva from the sublingual salivary glands of swiftlets [3] and are built as shelters, breeding areas, and perches [3]. The swiftlets live in caves along the coastlines of Southeast Asian countries, such as Indonesia, Malaysia, Thailand, Vietnam, and the Philippines [5]. The effects of human cultivation have caused many of the birds’ habitats to be modified into swiftlet farmhouses (SFHs), to which the swiftlet species *A. fuciphagus* is well adapted [6]. These farmhouses have been built in various locations, with some even close to human settlements. However, environmental and habitat changes have affected the diet of swiftlets, which can impact the nutritional content and potential food safety of EBN products.

In recent years, food safety issues concerning EBNs have become a significant concern for consumers and export destination countries. Food hazards related to EBNs that can potentially harm consumers include contamination with pathogenic bacteria, fungi [7], high nitrate and nitrite contents [8,9], and heavy metal contamination [7,10]. Here, the term “heavy metal” refers to any metallic element (such as cadmium [Cd], mercury [Hg], and lead [Pb]) or metalloid (e.g., arsenic [As]) with a relatively high density and toxicity even at low concentrations [11]. Cd, As, Hg, and Pb are toxic and harmful to human health [12]. Exposure to Cd can cause osteoporosis [13] and hypertension [14], while Hg exposure can cause neurological damage in pregnant women, fetuses, newborns, children, and adults [15] as well as impaired intelligence or behavioral dysfunction [16]. Pb toxicity can cause impaired intellectual and cognitive abilities that affect memory [17]. In contrast, As at high doses can cause cancer [18], Wilson’s disease, and even death [19]. Tin (Sn) can cause growth retardation, decreased food efficiency, anemia, inhibited iron absorption in the intestines, and degeneration of the liver [20].

Heavy metals in nature occur naturally and as a result of human activities, including industry, mining, and agriculture [21]. Heavy metals can accumulate in the environment; in soil, water, air [22], and contaminated vegetation [23]. In the environment, heavy metals can enter a swiftlet’s body and transfer to its products, including its nests [7]. Heavy metals can also enter via insects (food), water intake, air pollution, and SFH building materials. Before export, EBNs must be cleaned of feathers and dirt and washed.

This study aims to determine and compare the presence and concentrations of As, Hg, Pb, Cd, and Sn in EBNs originating from several primary EBN-producing Indonesian islands. Furthermore, the study also analyzes the effect of washing on the heavy metals present in EBNs.

## Materials and Methods

### Ethical approval

This study did not involve live swiftlets, so it did not require ethical approval.

### Research design

The study was conducted through a survey to detect and analyze the heavy metal levels in EBN samples from SHF originating from several main islands in Indonesia. Heavy metal levels in EBN samples from each SFH were measured before washing (raw-unclean EBN) and after washing (raw-clean EBN) washing using reverse osmosis (RO) water at the EBN processing site. The inductively coupled plasma mass spectrometry (ICP-MS) was used to detect the levels of the five heavy metals (As, Hg, Pb, Cd, and Sn).

### Study period and location

This research was conducted from August 2020 to April 2021. EBNs samples were collected from the islands of Java, Sumatera, Sulawesi, and Kalimantan. The samples were washed at the EBN processing plant, registered by the Ministry of Agriculture (Indonesia Agricultural Quarantine Agency/IAQA). Heavy metal levels were tested at the Quality Control Laboratory and Certification of Animal Products (QCLCAP) Bogor, West Java.

### Population and sample collection

**Figure-1:**
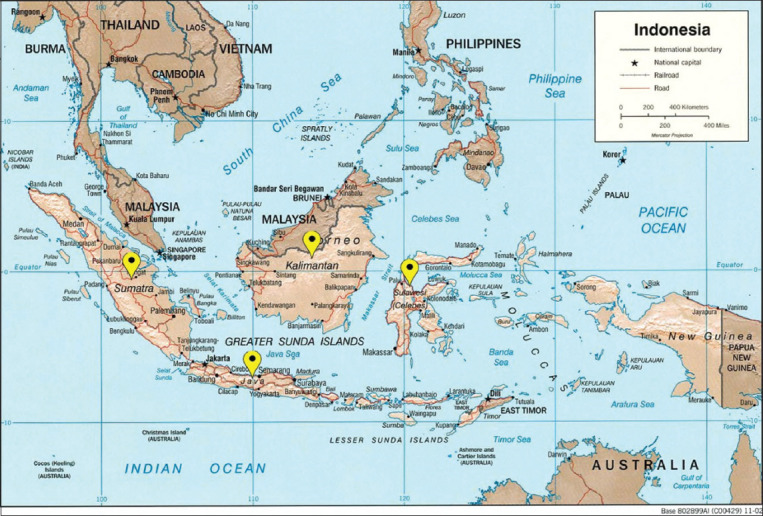
The four islands as the largest edible bird’s nest producers in Indonesia (Java, Sumatera, Sulawesi, and Kalimantan) [24].

Data on the number of SFHs in Indonesia have not been published previously, but it is estimated that more than 14,000 such units exist. However, in mid-2021, only 1335 SFH units had been registered with the country’s Ministry of Agriculture (IAQA 2021 August 16, personal communication). Registration is required for traceability purposes, particularly for EBNs that are exported to China. Java, Sumatra, Kalimantan, and Sulawesi are the largest EBN producers in Indonesia, and many SFHs can be found on these islands. The number of EBNs in Java, Sumatra, Sulawesi, and Kalimantan is estimated to be 1:6:3:10, based on information from the chairperson of the Farmers Association EBNs Nusantara (Thong 2020 August 4, personal communication). The islands from which the EBN samples were taken are shown in Figure-1 [24].

The study’s sample size was calculated using a mean-difference formula (μ1-μ2) in Open Epi (v.3.01) software (https://www.openepi.com/Menu/OE_Menu.htm), and data on Hg levels in EBNs in Malaysia were presented in terms of mean and standard deviation at a 95% confidence level [23]. A total of 44 SFHs were determined for the sampling of EBNs, and the number of SFH samples was allocated proportionally [25] to the islands included herein (i.e., 2, 13, 7, and 22 for Java, Sumatra, Sulawesi, and Kalimantan, respectively).

### Sample criteria

The criteria for the EBN samples collected from SFHs were as follows. The nests had to be white in color with a weight that ranged from 6 to 8 g/piece with a light feather category. Two pieces of freshly harvested EBNs were homogenized and analyzed as the study sample. Each sample from an SFH was divided into two parts; half of the EBN was washed, while the other half was not. Samples of EBNs were taken randomly from SFHs, regardless of their registration status.

### Washing method

The washing method used herein refers to the procedures used in the EBN processing plant. The EBN washing time was measured to ensure that it was the same for each sample, and the same group of samples was washed and treated in the same manner in the same processing plant. The processing plant was registered with the Agricultural Quarantine Agency of the Ministry of Agriculture for exporting EBNs, and it implemented hazard analysis critical control points.

All the unwashed EBN samples were cleaned of surface feathers and dirt. The unwashed halves of the EBNs (yet to be washed and cleaned) were grouped separately. The edges of the EBNs attached to the fins were scraped off with an iron grinder to remove any wood that had adhered to the nest. After removing the dirt, the EBN was ready to be washed.

The EBNs were washed using RO water for 10±2 s to remove minor impurities and soften their texture. The samples were dried using a hygienic food-processing type of tissue and were aerated for 150±5 min. Fine hairs and dirt were cleaned using food-grade stainless-steel tweezers and a soft brush. The samples were re-dried by aeration at room temperature for 120 min; then, the EBNs were shaped and curved. The curved samples were re-dried by aeration until the moisture level reached 10-12%. Then, the samples were ready for analysis using ICP-MS. Each step was conducted using different equipment to avoid cross-contamination.

### Detecting heavy metal concentrations using ICP-MS

Before conducting ICP-MS, a standard solution (Supelco Certipur^®^, Germany) was prepared for each of the five heavy metals considered in this study (i.e., As, Hg, Pb, Cd, and Sn) at a concentration of 1000 ppm, which was subsequently diluted to 1000 ppb. The standard series with levels of 1.0, 2.5, 5.0, 7.5, 10.0, 15.0, and 20 ppb was prepared in a 20-mL volumetric flask and calibrated using 5% nitric acid (HNO_3_) (Merck, Germany) [26].

Each EBN sample was pounded into a powder using a homogenizer. A powdered sample of 0.3 g was poured into a microwave digestion tube and spiked with the standard level of 5 g/L. Then, 2 mL of a 30% w/w hydrogen peroxide solution (Merck) was added to each tube, followed by the addition of 8 mL of 65% HNO_3_ (Merck). The sample was put into a vessel and digested by microwave digestion (Ethos One^®^-Milestone, Bergamo, Italy) for 4 h. After the digestion process, the sample was cooled and quantitatively transferred to a 50-mL volumetric flask. After calibration with demineralized water, the sample was ready for ICP-MS analysis (QTEGRA Thermo Scientific™ iCAP Q^®^, Germany) [27].

Before use, the ICP-MS machine was checked to ensure that the nebulizer’s argon (SII, Indonesia) flow was optimized and the gas pressure of the regulator was at the correct positions (8-9). The machine was calibrated for optimal resolution and sensitivity using a calibration blank comprising 1% nitric acid (Merck) in ultrapure water and a calibration standard (Spex CertiPrep^®^, USA) (based on the analyte to be determined), which was prepared by diluting the stock elements of the standard sample solutions in ranges from 1 to 20 ppb. Following calibration, ICP-MS was conducted to analyze the heavy metals in the EBN samples. As, Hg, Pb, Cd, and Sn [25] traces were measured using ICP-MS.

### Statistical analysis

The test results for the concentrations of heavy metals in the EBN samples were expressed in mg/kg (ppm), and the data were presented descriptively in terms of mean and standard deviation to observe the data distribution. Sample data on each heavy metal element were grouped according to their island of origin. The concentrations in each group were tested non-parametrically using the Kruskal–Wallis test and the Mann–Whitney U test to observe differences in the median of each island group [28]. The mean was considered statistically significant at p<0.05.

Comparisons of the heavy metal concentrations in the raw–unclean EBNs and the raw–clean EBNs are presented in terms of percentages. The data underwent non-parametric testing to observe the significance of the changes in heavy metal concentrations before and after washing. The correlation for each heavy metal element in the raw–unclean and raw–clean EBNs was calculated using Spearman’s rank correlation coefficient [28]. Data analysis was performed using Microsoft Excel 2019 (Microsoft, USA) and SPSS v.20 software (IBM Corp., NY, USA).

## Results

### Heavy metal concentrations in raw–unclean EBNs

The raw–unclean EBN samples were cleaned of physical contaminants, such as feathers and woodcuts, before being tested. The concentrations of heavy metals in the raw–unclean EBNs were calculated based on mean and standard deviation and were grouped according to their island of origin. The results of the heavy metal testing on the raw–unclean EBN samples are shown in Table-1.

**Table 1:** Level (mg/kg) of heavy metals in raw–unclean EBN originating from Java, Sumatera, Sulawesi, and Kalimantan.

Heavy metal elements	Islands Origin			

	Java	Sumatera	Sulawesi	Kalimantan
Arsenic	0.0289±0.0076^a^	0.0177±0.0225^a^	0.0187±0.0212^a^	0.0110±0.0118^a^
Mercury	0.0329±0.0464^a^	0.0015±0.0046^a^	0.0000±0.0000^a^	0.0080±0.0177^a^
Lead	1.0299±0.2668^b^	0.6323±0.2424^b^	0.3512±0.3658^b^	0.0764±0.2721^c^
Cadmium	0.0288±0.0069^d^	0.0311±0.0137^d^	0.0199±0.0155^d^	0.0021±0.0063^e^
Tin	0.0565±0.0787^a^	0.0322±0.0562^a^	0.0509±0.0547^a^	0.0811±0.1761^a^

Data value is mean±standard deviation in dry EBN. EBN=Edible bird’s nest

Levels of As, Hg, Pb, Cd, and Sn were detected in most of the raw–unclean EBN samples from all the included islands; however, Hg was not detected for Sulawesi in any of the raw–unclean EBN samples. Most of the heavy metal elements originating from Java were detected at higher levels compared with the other islands. The levels of Pb and Cd in the raw–unclean EBNs originating from Kalimantan were significantly lower compared with the remaining islands (p<0.05).

### Heavy metal concentrations in raw–clean EBNs

Table-2 presents the test results for the heavy metal concentrations in the raw–clean EBN samples washed and cleaned at the EBN processing plant. Compared with the raw–unclean EBN samples, all heavy metal concentrations decreased in the raw–clean EBN samples sourced from all the islands. The levels of As and Pb from the samples from Java and Sumatra were significantly higher (p<0.05) than the EBNs from Sulawesi and Kalimantan. The level of Cd in the samples from Kalimantan was significantly lower compared with the remaining islands (p<0.05). Pb and Cd were not detected in the EBNs from Kalimantan after washing.

**Table 2:** Level (mg/kg) of heavy metals in raw-clean EBN originating from Java, Sumatera, Sulawesi, and Kalimantan.

Heavy metal elements	Islands Origin			

	Java	Sumatera	Sulawesi	Kalimantan
Arsenic	0.0145±0.0060^a^	0.0061±0.0036^a^	0.0009±0.0008^b^	0.0049±0.0052^b^
Mercury	0.0000±0.0000^c^	0.0001±0.0003^c^	0.0000±0.0000^c^	0.0035±0.0112^c^
Lead	0.3146±0.4449^def^	0.0898±0.1732^def^	0.0122±0.0323^f^	0.0000±0.0000^f^
Cadmium	0.0069±0.0098^g^	0.0023±0.0049^g^	0.0004±0.0009^g^	0.0000±0.0000^h^
Tin	0.0152±0.0211^c^	0.0061±0.0116^c^	0.0179±0.0282^c^	0.0122±0.0267^c^

Data value is mean±standard deviation in dry EBN. EBN=Edible bird’s nest

### The effect of washing on heavy metal concentrations in EBNs

**Figure-2:**
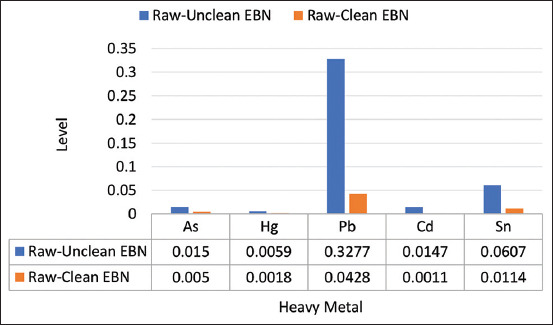
Comparison of heavy metal levels (mg/kg) in raw–unclean and raw–clean edible bird’s nest.

The average presence of each heavy metal element was calculated for the raw–unclean and raw–clean EBN samples. A decrease in the concentrations of all the heavy metals occurred after the EBNs had been washed. The heavy metal concentrations in the gross EBNs compared with the raw–clean EBNs are presented in Figure-2, which shows a decrease in the levels of all the heavy metal elements in the raw–clean EBNs. There was a significant decrease (p<0.05) in the concentrations of As, Pb, Cd, and Sn in the raw–clean EBNs.

**Figure-3:**
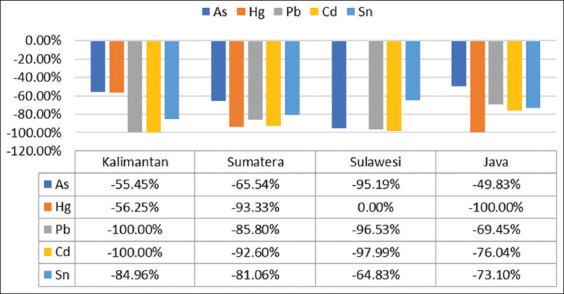
The percentage decrease in the level of heavy metals in the raw-unclean edible bird’s nest (EBN) minus the raw-clean EBN.

The EBNs were washed with RO water reflected decreased levels of heavy metals. The least reduction of heavy metals in As from Java is 49.83% compared with raw–unclean EBN. The highest reduction in heavy metals was found for Pb and Cd in the samples from Kalimantan (as high as 100% from the initial level). The percentage changes in the concentrations of heavy metals in the raw–clean EBN samples are shown in Figure-3.

### Heavy metal correlations in raw–unclean and raw–clean EBNs

Spearman’s correlation test for the concentration of several heavy metals in the raw–unclean and raw–clean EBNs showed a strong correlation between Pb and Cd. The correlation coefficient between these two heavy metals in the raw–unclean EBN samples was 0.929, while it was 0.922 in the raw–clean EBNs. Table-3 presents the correlation test results between each heavy metal element in the raw–unclean and raw–clean EBN samples.

**Table 3:** Spearman correlation coefficient between heavy metals in raw–unclean EBN and raw–clean EBN.

Heavy metals combination	Raw-unclean EBN		Raw-clean EBN	
	
	Correlation coefficient	p-value	Correlation coefficient	p-value
As-Hg	−0.074	0.634	−0.162	0.294
As-Pb	0.076	0.624	0.210	0.172
As-Cd	0.121	0.435	0.116	0.454
As-Sn	0.184	0.231	0.257	0.092
Hg-Pb	0.000	0.999	−0.141	0.360
Hg-Cd	−0.136	0.377	−0.157	0.309
Hg-Sn	0.212	0.167	−0.084	0.586
Pb-Cd	0.929**	0.000	0.922**	0.000
Pb-Sn	0.041	0.793	0.003	0.982
Cd-Sn	0.010	0.947	−0.050	0.747

*There is a significant relationship (mean) at the level of 0.05.

** There is a very significant relationship (mean) at the level of 0.01. As=Arsenic, Hg=Mercury, Pd=Lead, Cd=Cadmium, Sn=Tin. EBN=Edible bird’s nest

## Discussion

### Heavy metal concentrations in raw–unclean EBNs

The raw–unclean EBNs that originated from Java had the highest levels of As, Hg, and Pb. Human activities are increasing in areas with high population densities. In Java, this is as high as 56.10% (151.6 million people), followed by Sumatera, Sulawesi, and Kalimantan, respectively, at 21.68% (58.6 million people), 7.36% (19.9 million people), and 5.54% (15.0 million people), while the remaining population lives on other islands [29]. Industrial, household and agricultural activities cause diverse environmental pollution [30], including in the feeding environments of swiftlets. Anthropogenic sources of As include metal smelting industries, household combustion, power plants, As fungicides, insecticides, herbicides, algicides, wood preservatives, and plant and animal growth stimulants [31]. Anthropogenic sources of Hg include mining, incineration, smelting emissions, and fungicides [32].

The concentrations of As, Hg, and Sn in raw–unclean EBNs were not significantly different between the main EBN-producing islands in Indonesia. This result differed from research conducted by Quek *et al*. [23], which found that As and Hg showed the most significant (p<0.05) differentiations related to their geographic origins in Malaysia (between Peninsular Malaysia and East Malaysia). The results of research conducted by Chen *et al*. [7] in Kluang (Malaysia) also found Hg levels up to 70,180 ppb in raw–unclean EBN samples.

The concentrations of Pb and Cd in the raw–unclean EBN samples from Java, Sumatra, and Sulawesi were higher than in the raw–unclean EBNs from Kalimantan (p<0.05). The accumulation of these heavy metals indicates the presence of air [33] and water pollution [34,35], which can be transmitted through food [31,36] and water [37]. Those pollutants are the products of industrial and agricultural activities [38]. The high levels of heavy metals found in the raw–unclean EBNs from Java were the result of the presence of many industrial and community activities on the island compared with other islands in the region.

Pb and Cd levels in the raw–unclean EBN samples from Kalimantan were lower than those for the other islands, which is indicative of being food safe. In addition, the lower levels indicated a healthier environment and good soil, water, and air quality related to human activities [30].

### Heavy metal concentrations in raw–clean EBNs

The maximum limit for heavy metals in raw–unclean and raw–clean EBNs in Indonesia is not currently regulated. However, there are standards related to the maximum heavy metal limits in Malaysia and China for raw–clean EBNs, as shown in Table-4 [39].

**Table 4:** Standards for maximum heavy metal limits in Indonesia, Malaysia, and China for raw–clean EBN [39].

Heavy metals	Indonesia	Malaysia	China
As	N/A	≤1 mg/kg	≤1 mg/kg
Hg	N/A	≤0.05 mg/kg	≤0.05 mg/kg
Pb	N/A	≤2 mg/kg	≤2 mg/kg
Cd	N/A	≤1 mg/kg	≤1 mg/kg
Sn	N/A	N/A	N/A

N/A=Not available. As=Arsenic, Hg=Mercury, Pd=Lead, Cd=Cadmium, Sn=Tin. EBN=Edible bird’s nest

All heavy metal concentrations in the raw–clean EBNs taken from the islands included in this study were below the maximum limits set by China and Malaysia. In a study by Tan et al. [40], the results for seven samples of raw–clean EBNs from six different regions in Malaysia showed values below China’s standards, based on an in-house analysis method conducted according to AOAC 999.11. Hg and Cd levels in raw–clean EBNs were the lowest compared with other heavy metal elements. These findings are almost identical to those presented by Hun et al. [41], who reported no detectable Hg or Cd in raw–clean EBNs; however, both As and Pb were detected.

### Effect of washing on heavy metal concentrations in EBNs

The concentrations of all heavy metal elements decreased after the EBNs were washed and cleaned. The number of external contaminants and the innate residues from swiftlets influenced heavy metal levels in the raw–unclean EBNs. The decrease in heavy metal concentrations in the raw–clean EBNs was due to dissolved and waste water that had been used for washing. In addition, heavy metal levels decreased following the loss of external contaminants, that is, the removal of dirt and hair. Raw–unclean EBNs are rich in particulate contaminants, for example, organic matter, which increases the heavy metal content. The presence of organic particles can also cause the bioaccumulation of heavy metal elements [42].

The washing procedure significantly reduced the levels of As, Pb, Cd, and Sn (p<0.05). The results of this study are consistent with research conducted by Borghesi *et al*. [42], which showed that washing bird feathers from EBNs led to a significant decrease in the concentrations of As, Pb, and Cd.

Hg levels decreased in the raw–clean EBNs (statistically but not significantly different), presumably because the Hg level had a minimal value in the raw–unclean EBNs. Borghesi *et al*. [42] found that the Hg level in bird feathers was not significantly reduced after washing, and Hg levels in feathers were also not affected by washing treatments [43]. This contrasted with the results of the present study, which indicated a reduction in Hg levels after washing the raw–unclean EBNs. This study of heavy metals in swiftlets provides an overview of the effectiveness of washing to decrease the concentration of heavy metals in EBN and the types of metals involved.

Indonesia has gradually banned Hg in mining under Law no. 11 of 2017 concerning the ratification of the Minamata Convention on Hg. This prohibition aims to protect the Indonesian environment against the hazards of Hg. The Hg levels in the EBNs sourced from all the islands in the current study were low, and they were undetectable in swiftlets from Sulawesi.

### Correlation between heavy metals in raw–unclean and raw–clean EBNs

The presence of one heavy metal element in swiftlets can be related to other such metal elements, as indicated by the correlation coefficient. The level of heavy metals in Pb and Cd was significantly correlated with both raw–unclean and raw–clean EBNs (Table-3). The correlation between Pb and Cd indicated that a link existed between these metals during the contamination [44] of EBNs in SFHs.

The washing method that was used in this study referred to the procedures of a processing plant registered by the Ministry of Agriculture (the Agricultural Quarantine Agency), Indonesia. The quality of the produced EBNs described the products on the market. This study used samples from Indonesia’s four largest EBN-producing islands, all of which have different geographical ecosystem characteristics. Accordingly, the results are representative of each island’s environmental conditions and describe the situation related to EBNs in Indonesia. Research related to analyzing the presence of heavy metals in EBNs and evaluating the effectiveness of washing to reduce these in Indonesia has not been conducted to date.

## Conclusion

Heavy metals (As, Hg, Pb, Cd, and Sn) were detected at low concentrations in most of the raw–unclean EBNs originating from the islands of Java, Sumatra, Sulawesi, and Kalimantan. The concentrations of all the heavy metals tested in the EBN samples from Java were higher than the other islands. Pb contamination was closely related to Cd in the raw–unclean and raw–clean EBNs. The concentrations of all heavy metals in the raw–unclean EBNs decreased significantly after washing.

## Authors’ Contributions

DSW, HL, MBS, and CB: Designed the study. DSW: Carried out sample collection and testing. DSW: Carried out the washing treatment. HL, DT, MBS, and CB: Supervised the study. DSW and CB: Performed the statistical analysis. DSW: Drafted the manuscript. HL, MBS, CB, and DT: Reviewed and edited the manuscript. All authors read and approved the final manuscript.

## References

[1] Huang X, Li Z, Xiaobo Z, Shi J, Tahir H.E, Xu Y, Zhai Y, Hu X (2020). Geographical origin discrimination of edible bird's nests using smart handheld device based on colorimetric sensor array. J. Food Meas. Charact.

[2] Badan Pusat Statistik (2021). Ekspor Sarang Burung Menurut Negara Tujuan Utama, 2012-2020.

[3] Koon L.C, Cranbrook E (2002). Swiftlets of Borneo-Builders of Edible Nests. Natural History Publication (Borneo) SDN.

[4] Wan Khairy W.I, Yusof M.N.A, Lian C.J, Yaacob M.R, Jayara V.K (2019). Megaderma lyra Predation on *Aerodramus* spp. Impact on Malaysian Bird Nest Industry.

[5] Saengkrajang W, Matan N, Matan N (2013). Nutritional composition of the farmed edible bird's nest (*Collocalia fuciphaga*) in Thailand. J. Food Compost. Anal.

[6] Fujita M, Leh C (2020). The Feeding Ecology of Edible-Nest Swiftlets in a Modified Landscape in Sarawak. In Anthropogenic Tropical Forests.

[7] Chen J.X.J, Lim P.K.C, Wong S.F, Mak J.W (2014). Determination of the presence and level of heavy metals and other elements in raw and commercial edible bird nests. Mal. J. Nutr.

[8] Paydar M, Wong Y.L, Wong W.F, Hamdi O.A.A, Kadir N.A, Looi C.Y (2013). Prevalence of nitrite and nitrate contents and its eﬀect on edible bird nest's colour. J. Food Sci.

[9] Quek M.C, Chin N.L, Aniza Y.Y, Tan S.W, Law C.L (2015). Preliminary nitrite, nitrate, and colour analysis of Malaysian edible bird's nest. Inf. Process. Agric.

[10] Duffus J.H (2002). Heavy metals a meaningless term?. Pure Appl. Chem.

[11] Lee T.H, Waseem A.W, Tan E.T.T, Nur A.A, Yong L.L, Ramlan A.A (2015). Investigations into the physicochemical, biochemical and antibacterial properties of edible bird's nest. J. Chem. Pharm. Res.

[12] Ha E, Basu N, Bose-O'Reilly S, Dórea J.G, Mcsorley E, Sakamoto M, Chan H.M (2016). Current progress on understanding the impact of mercury on human health. Environ. Res.

[13] Honda R, Tsuritani I, Noborisaka Y, Suzuki H, Ishizaki M, Yamada Y (2003). Urinary cadmium excretion is correlated with calcaneal bone mass in Japanese women living in an urban area. Environ. Res.

[14] Kopp S.J, Glonek T, Perry H.M, Erlanger M, Perry E.F (1982). Cardiovascular actions of cadmium at environmental exposure levels. Science.

[15] Debes F, Budtz-Jrgensen E, Weihe P, White R.F, Grandjean P (2006). Impact of prenatal methylmercury exposure on neurobehavioral function at age 14 years. Neurotoxicol. Teratol.

[16] Yorifuji T, Tsuda T, Inoue S, Takao S, Harada M (2011). Long-term exposure to methylmercury and psychiatric symptoms in residents of Minamata, Japan. Environ. Int.

[17] Koller K, Brown T, Spurgeon A, Levy L (2004). Recent developments in low-level lead exposure and intellectual impairment in children. Environ. Health Perspect.

[18] Jarup L (2003). Hazards of heavy metal contamination. Br. Med. Bull.

[19] Huster D, Purnat T.D, Burkhead J.L, Ralle M, Fiehn O, Stuckert F, Olson N.E, Teupser D, Lutsenko S (2007). High copper selectively alters lipid metabolism and cell cycle machinery in the mouse model of Wilson disease. J. Biol. Chem.

[20] Cima F (2011). Tin:Environmental pollution and health effects. Encyclopedia Environmental Health.

[21] Ali H, Khan E, Ilahi I (2019). Environmental chemistry and ecotoxicology of hazardous heavy metals:Environmental persistence, toxicity, and bioaccumulation. J. Chem.

[22] Alengebawy A, Abdelkhalek S.T, Qureshi S.R, Wang M.Q (2021). Heavy metals and pesticides toxicity in agricultural soil and plants:Ecological risks and human health implications. Toxics.

[23] Quek M.C, Chin N.L, Yusof Y.A, Law C.L, Tan S.W (2018). Characterization of edible bird's nest of different production, species and geographical origins using nutritional composition, physicochemical properties and antioxidant activities. Food Res. Int.

[24] Chik R (2018). Gambar Peta Indonesia Lengkap Dengan Skala.

[25] Cameron A (1999). Survey Toolbox for Livestock Disease:A Practical Manual and Software Package for Active Surveillance in Developing Countries.

[26] Association of Official Agricultural Chemists (2012). Heavy Metals in Food Inductively Coupled Plasma-Mass Spectrometry.

[27] Milestone (2011). PRO High Versatile Rotor Application Book.

[28] Lang T.A, Altman D.G (2015). Basic statistical reporting for articles published in biomedical journals:The “statistical analyses and methods in the published literature”or the SAMPL guidelines. Int. J. Nurs. Stud.

[29] Badan Pusat Statistik (2021). Hasil Sensus Penduduk 2020. Badan Pusat Statistik.

[30] Caggiano R, Sabia S, D'Emilio M, Macchiato M, Anastasio A, Ragosta M, Paino S (2005). Metal levels in fodder, milk, dairy products, and tissues sampled in ovine farms of Southern Italy. Int. J. Environ. Res.

[31] Matta G, Gjyli L (2016). Mercury, lead and arsenic:Impact on environment and human health. J. Chem. Pharm. Sci.

[32] Chiarelli R, Roccheri M.C (2015). Marine invertebrates as bioindicators of heavy metal pollution. Open J. Metal.

[33] Dmuchowski W, Gozdowski D, Baczewska A.H (2011). Comparison of four bioindication methods for assessing the degree of environmental lead and cadmium pollution. J. Hazard. Mater.

[34] Burger J, Gochfeld M (1993). Lead and cadmium accumulation in eggs and fledgling seabirds in the New York bight. Environ. Toxicol. Chem.

[35] Abadin H, Ashizawa A, Stevens Y.W, Llados F, Dia Monteiro D.G, Sage G, Citra M, Quinones A, Bosch S.J, Swarts S.G (2007). Toxicological Profile for Lead.

[36] Dubois M, Hare L (2009). Subcellular distribution of cadmium in two aquatic invertebrates:Change over time and relationship to Cd assimilation and loss by a predatory insect. Environ. Toxicol. Chem.

[37] Burger J, Gochfeld M, Sullivan K, Irons D (2007). Mercury, arsenic, cadmium, chromium lead, and selenium in feathers of pigeon guillemots (*Cepphus columba*) from Prince William Sound and the Aleutian Islands of Alaska. Sci. Total Environ.

[38] Vizuete J, Pérez-López M, Míguez-Santiyán M.P, Hernández-Moreno D (2018). Mercury (Hg), lead (Pb), cadmium (Cd), selenium (Se), and arsenic (As) in liver, kidney, and feathers of gulls a review. Rev. Environ. Contam. Toxicol.

[39] Yeo B.H, Tang T.K, Wong S.F, Tan C.P, Wang Y, Cheong L.Z, Lai O.M (2021). Potential residual contaminants in edible bird's nest. Front. Pharmacol.

[40] Tan S.N, Sani D, Lim C.W, Ideris A, Stanslas J, Lim C.T.S (2020). Proximate analysis and safety profile of farmed edible bird's nest in Malaysia and its effect on cancer cells. Evid. Based Complement. Altern. Med.

[41] Hun L.T, Wani W.A, Tjih E.T.T, Adnan N.A, Le Ling Y, Aziz R.A (2015). Investigations into the physicochemical, biochemical, and antibacterial properties of edible bird's nest. J. Chem. Pharm. Res.

[42] Borghesi F, Dinelli E, Migani F, Bechet A, RendónMartos M, Amat J.A, Sommer S, Gillingham M.A (2017). Assessing environmental pollution in birds:A new methodological approach for interpreting bioaccumulation of trace elements in feather shafts using geochemical sediment data. Methods Ecol. Evol.

[43] Appelquist H, Asbirk S, Drabæk I (1984). Mercury monitoring:Mercury stability in bird feathers. Mar. Pollut. Bull.

[44] Aziz B, Zubair M, Irshad N, Ahmad K.S, Mahmood M, Tahir M.M, Shah K.H, Shaheen A (2021). Biomonitoring of toxic metals in feathers of birds from North-Eastern Pakistan. Bull. Environ. Contam. Toxicol.

